# Prediction of Compound Synthesis Accessibility Based on Reaction Knowledge Graph

**DOI:** 10.3390/molecules27031039

**Published:** 2022-02-03

**Authors:** Baiqing Li, Hongming Chen

**Affiliations:** 1 Guangdong Provincial Key Laboratory of Laboratory Animals, Guangdong Laboratory Animals Monitoring Institute, Guangzhou 510663, China; li_baiqing@grmh-gdl.cn; 2 State Key Laboratory of Respiratory Disease, Guangzhou Institutes of Biomedicine and Health, Chinese Academy of Sciences, Guangzhou 510530, China; 3 Bioland Laboratory (Guangzhou Regenerative Medicine and Health-Guangdong Laboratory), Guangzhou 510530, China; 4 Guangzhou Laboratory, Guangzhou International Bio Island, No. 9 XinDaoHuanBei Road, Guangzhou 510005, China

**Keywords:** synthesis accessibility, reaction knowledge graph, graph model

## Abstract

With the increasing application of deep-learning-based generative models for de novo molecule design, the quantitative estimation of molecular synthetic accessibility (SA) has become a crucial factor for prioritizing the structures generated from generative models. It is also useful for helping in the prioritization of hit/lead compounds and guiding retrosynthesis analysis. In this study, based on the USPTO and Pistachio reaction datasets, a chemical reaction network was constructed for the identification of the shortest reaction paths (SRP) needed to synthesize compounds, and different SRP cut-offs were then used as the threshold to distinguish a organic compound as either an easy-to-synthesize (ES) or hard-to-synthesize (HS) class. Two synthesis accessibility models (DNN-ECFP model and graph-based CMPNN model) were built using deep learning/machine learning algorithms. Compared to other existing synthesis accessibility scoring schemes, such as SYBA, SCScore, and SAScore, our results show that CMPNN (ROC AUC: 0.791) performs better than SYBA (ROC AUC: 0.76), albeit marginally, and outperforms SAScore and SCScore. Our prediction models based on historical reaction knowledge could be a potential tool for estimating molecule SA.

## 1. Introduction

The fact that the drug-like chemical space [1,2,3] is around 10^60^–10^100^ makes the process of finding a compound that simultaneously satisfies the plethora of criteria, such as bioactivity, drug metabolism and pharmacokinetic (DMPK) profile, as well as synthetic accessibility, as difficult as finding a needle in a haystack [4,5,6]. Hence, both medicinal and computational chemists attempted to develop approaches to efficiently explore chemical space to identify the compounds with desirable pharmacological activities as well as ADMET properties [7,8,9,10,11]. Among these efforts, the virtual-library-based de novo molecule design method represents an important computational paradigm [12,13,14,15]. The application of deep generative modelling for de novo molecule design has emerged in recent years. One major benefit of the generative model method is that it can exhaustively explore a much larger chemical space compared with virtual-library-based methods. However, one big hurdle for structural generation in the generative model is ensuring that the generated compounds are synthetically accessible [16]. Ideally, the compounds designed by those generative models should be able to be synthesized within a few synthesis steps. 

Strictly speaking, synthetic accessibility (SA) and molecular complexity are different concepts. The definition of molecule complexity is context-dependent and sometimes ambiguous, as there is no objective way to quantify the “complexity” of a molecule. Usually, people regard molecules that possess multiple function groups, complex ring systems or multiple chiral centre as complex structures due to the difficulties with synthesis [17]. SA is, to some extent, related to molecular complexity. However, sometimes, structural complexity is not necessary equivalent to the SA [18], when the availability of the starting materials is taken into account [19,20]. For example, the total synthesis of a steroid is a tedious and challenging task; however, if starting from readily prepared intermediates such as cholesterol, the synthesis might only require very few reaction steps [21]. Therefore, the molecular-complexity-based metrics could underestimate the SA of the molecules, which can easily be synthesized from already-existing precursors [19,20]. Nevertheless, in most SA scores/models, it was molecular complexity that was actually modeled and SA served as a synonym of molecular complexity. 

A simple SA assessment can be conducted by simply calculating some physicochemical properties such as number of atoms, bonds, rings and some unconventional hard-to-synthesis motifs, such as stereo-centre and macrocycles, etc. Boda et al. [17] proposed a scoring method for rapidly evaluating SA based on compound similarity to available starting materials and specific bonds, where a structure can be decomposed to obtain simpler fragments. A linear regression analysis was carried out to correlate different structural components to the accessibility scores given by medicinal chemists. An SAScore [22] was developed based on the frequency analysis of molecular ECFP4 [23] fragment occurrence in PubChem database. This proved to be a useful tool in many cheminformatics applications [24,25,26,27]. The method correlates the SA of a molecule to the fragment’s frequency of occurrence. Each fragment is assigned a numerical SA score. The higher the fragment’s frequency of occurrence in the PubChem database [28], the greater its SA score. 

In the absence of synthesis yield, a more realistic solution for estimating SA is to take the reaction route complexity [29] into account, which means that the higher the number of reaction steps needed to synthesize the compound, the lower the synthesis accessibility of the compound. Although there is some domain-specific knowledge of what a good or reasonable synthetic route should be, in general, the synthesis complexity of compound becomes larger when more synthesis steps are needed [30,31]. According to this principle, an SCScore [21] was recently developed based on one simple premise: on average, reaction products are synthetically more complex than their corresponding reactants. By building 22 million reactant–product pairs from the commercial Reaxys database [32], a deep feed-forward neural network was trained to assign a synthetic accessibility score between 1 and 5. The main idea of the SCScore was to learn an SA score that correlates with the number of reaction steps. However, merely taking isolated reactant-product pairs into account, without considering the relationship between compounds across different pairs will probably mean that the method is not general enough to characterize the SA. SYBA [18], another recent method of SA assessment, is a fragment-based method for classifying organic compounds into either an easy-to-synthesis (ES) or hard-to-synthesis (HS) class. To quantify this, a Bernoulli-naïve Bayes classifier was trained on ES molecules available from ZINC15 [33] and HS molecules generated using the Nonpher [34] algorithm. However, SYBA has the same problems as SCScore in terms of the construction of a dataset as one-to-one (ES and HS) pairs; both methods lack a systematic comparison in a large chemical reaction database. It is worth mentioning that the recently published RAscore [35], a feed-forward neural network classifier, based on the AI-driven computer-aided synthesis planning (CASP) tool—AiZynthFinder [36]—could propose synthetic routes for a particular compound, and assign a synthetic accessibility value based on predicted synthesis steps. In this case, the reaction data were implicitly taken into account. In terms of dataset selection, the ES dataset was generated by randomly sampling 200,000 compounds from ChEMBL, and the HS dataset was collected from the GDB17 database [37]. 

Gryzbowski et al. [38] reported their work on constructing a reaction knowledge graph, aka a network of chemistry (NOC), where a large amount of compounds are inter-connected as either reactants or products. In the current study, a similar knowledge graph based on a reaction dataset of USPTO [39] and Pistachio [40] was constructed. According to the direction of the connected edges, nodes in the network were classified into two types of node: the node serving only as a reactant (i.e., starting materials) and the normal node, which can serve as either the reactant or product. The edge distance between the normal node and the reactant-only node in the graph is used to represent the possible reaction steps for synthesizing a compound. According to the shortest reaction paths (SRP) in the reaction network, an organic compound can be labeled as either ES or HS. 

Instead of constructing a dataset in the pairwise manner, such as SCScore and SYBA, a dataset based on the existing reaction evidence in the reaction knowledge graph can be curated. Different SRP thresholds were then used to distinguish an organic compound as either the easy-to-synthesize (ES) or hard-to-synthesize (HS) class. Various deep-learning-based classification models, which include the graph neural network (CPMNN [41]) as well as the fully connected feed-forward neural network, were built to predict a compound’s SA. A comparison between our deep learning models and the existing SA scoring functions, such as SYBA, SCScore and SAScore, was also carried out. 

## 2. Methods

### 2.1. Reaction Dataset

**Figure 1:**
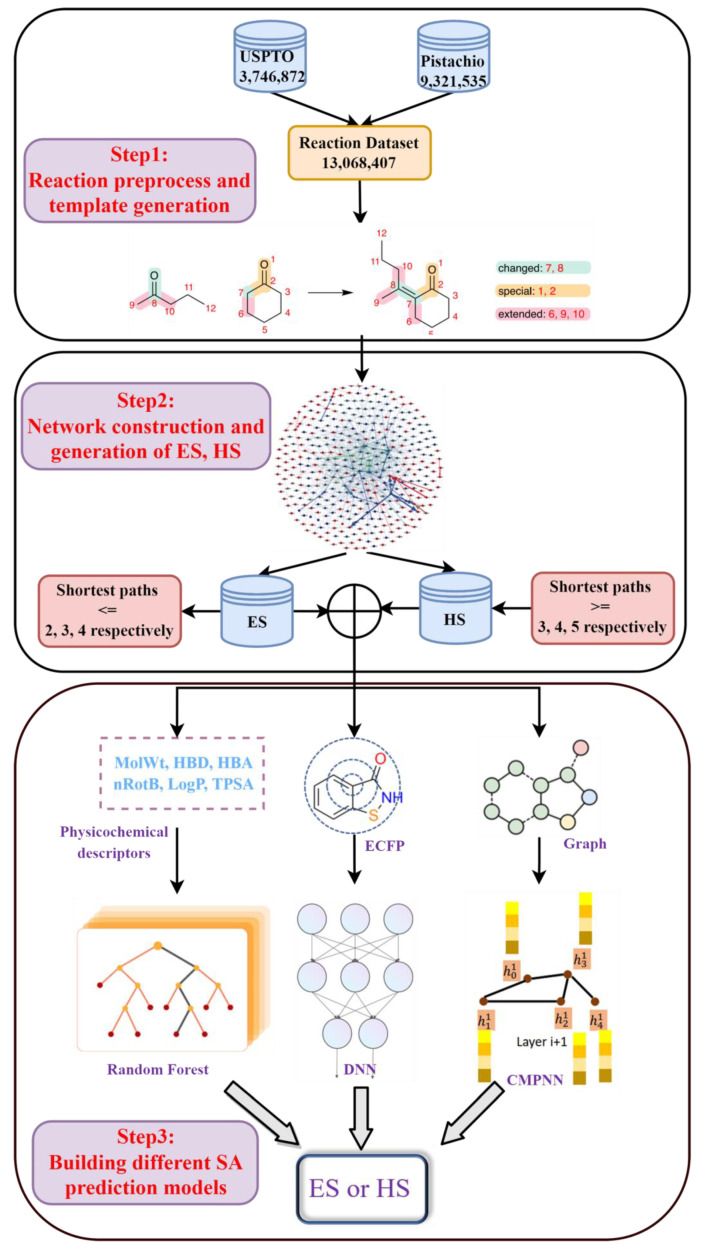
The workflow of chemical reaction processing. It was divided into three steps: (1) reaction preprocess and template generation; (2) network construction and generation of ES, HS datasets; (3) building different SA prediction models.

The reaction dataset from the publicly available United States Patent Office extracts (USPTO [39], 3,746,872 items in total), ranging from 1976 to 2016, and the commercially available Pistachio [40] database (9,321,535 items in total), were used in the current study. After removing duplicates, all reactions were atom-mapped and classified using Filbert [42] and HazELNut [43] program provided by NextMove, and 12,985,183 reaction items remained. The dataset was then cleaned using the predefined filtering criteria described by Thakkar et al. [44] to remove some undesirable reactions (such as incomplete reactions, or reactions which cannot generate reaction template [44,45]). Here, the reaction template refers to the subgraph pattern, which describes the maximal common connectivity changes when reactants are transformed to products in a class of reaction, and it is basically an abstract representation of a class of reaction [45]. In total, 9,041,882 valid reaction items were obtained. This dataset was further processed with the workflow shown in Figure 1 for template extraction, knowledge graph generation and the building of various predictive models. 

### 2.2. Template Extraction and Role Designation

**Figure 2:**
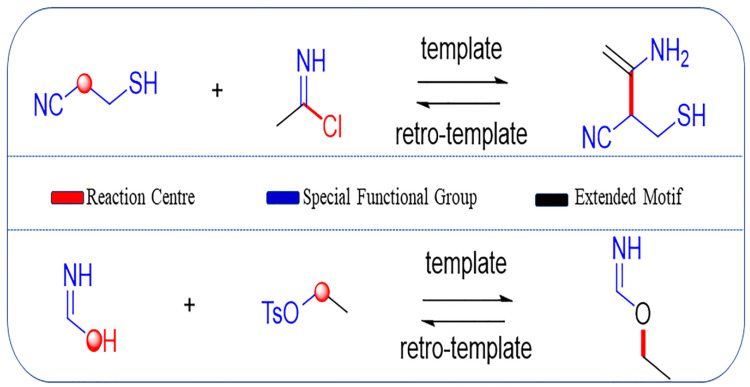
Examples of template extraction, in which colored sites are added to the reaction template. The red part refers to the reaction centers where the reaction occurs; blue part refers to the special functional groups around the reaction center; black part corresponds to the extended motif, which is the carbon atoms within a certain distance to the reaction center.

Coley et al. [45,46] recently reported the development of a toolkit, RDChiral [45], for reaction template extraction. This toolkit is not only able to recognize the immediate neighborhood of reaction centers (red part in Figure 2), but also the required extended environment, including special functional groups (blue part in Figure 2) and neighboring carbon atoms as the extended motif (black part in Figure 2), based on a user-defined radius (default value radius = 1 used here). It is worth mentioning that Thakkar et al. [44] added 70 additional functional groups and protecting groups, besides the original 75 special groups that were included in RDChiral. The valid reaction set mentioned above was subsequently processed using RDKit and RDChiral for template extraction. In total, 7,466,854 reaction templates were produced. 

**Figure 3:**
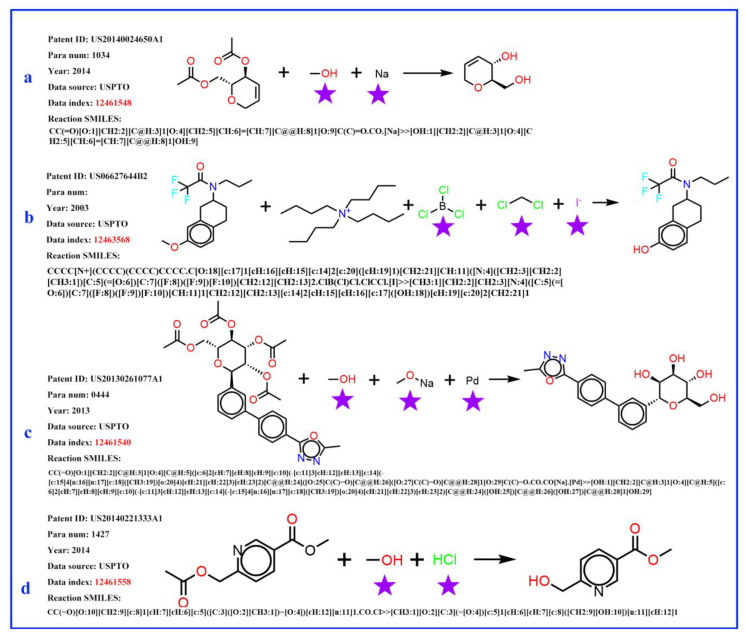
Four examples of original flawed chemical reaction records. The obvious error is marked by a purple pentagram. (**a**) sodium (Na) does not belong to reactants, the methanol serves as a solvent; (**b**) the iodide ion does not belong to this reaction, the methylene dichloride serves as a solvent role; boron tri-chloride and the ammonium ion do not contribute mass to the product and do not belong to the reactant; (**c**) Pd is catalyst, the sodium methanolate serves as a base, and the methyl alcohol is solvent; (**d**) the methyl alcohol is solvent, the HCl provides an acidic environment and is assigned to the reagent role.

**Figure 4:**
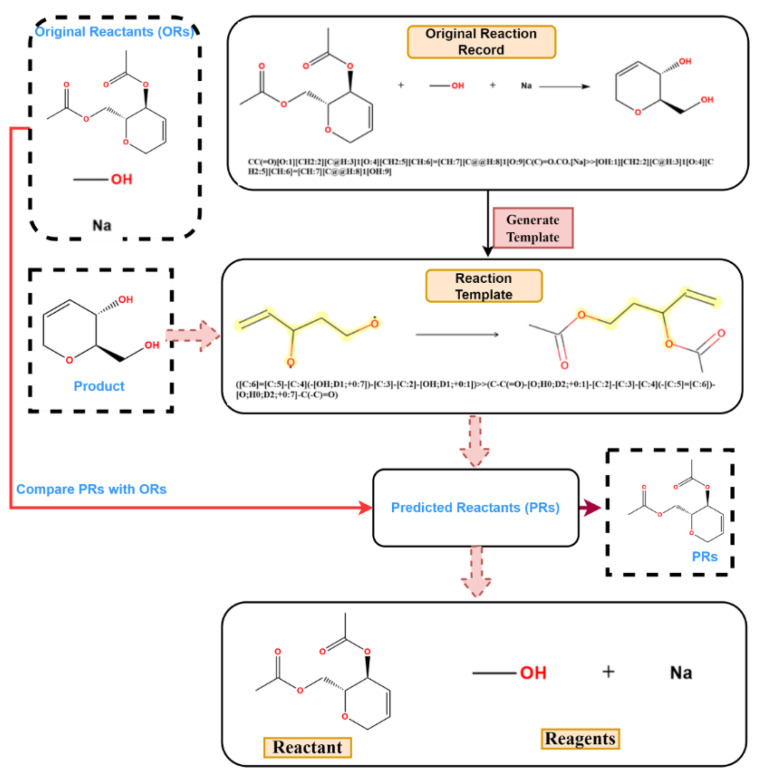
The process of role designation: (1) rearrange ORs; (2) generate inverse reaction templates; (3) match original product with inverse templates and get PRs; (4) compare PRs with ORs.

It was found that, in the reaction records processed in the previous procedure, the reagent, part of the SMILES reaction was often misplaced into the reactant part. For example, in Figure 3a, it was obviously wrong to put the sodium (Na) into the reactant list and the methanol should be solvent; in Figure 3b, the iodide ion cannot exist in this reaction, and the methylene dichloride should be solvent. The exact role of boron tri-chloride is unknown, but it is clear that boron tri-chloride and the ammonium ion do not contribute mass to the product and do not belong to the reactant. In Figure 3c, Pd is the catalyst, the sodium methanolate serves as the base, and the methyl alcohol is the solvent; in Figure 3d, the methyl alcohol is the solvent, while the HCl provides an acidic environment and is assigned to the reagent. In these problematic reaction records, the reagents/solvents in the SMILES reaction were mixed with the reactants, and this could cause troubles when identifying the correct reactant structures in the following reaction network creation step. However, this misplacement did not affect the template extraction process, as all non-product parts were treated equally in the operation. The reaction templates extracted above were then used to identify the reaction roles (mainly the reactants and reagents). A Knime [47] workflow was developed to clean up the reactant list of each reaction and the following steps were carried out to identify reagent components (as depicted in Figure 4): (1) Before extracting templates, all reagent components (solvent and catalyst) of the original reaction records were treated as reactants and formed original reactants (ORs). (2) Template extraction procedure was carried out based on the reorganized reaction SMILES, and both forward and inverse reaction templates were generated. The products were then put into the inverse reaction template to generate the predicted reactants (PRs) using RDChiral [17] functions. (3) For each reaction record, the generated PRs were then compared with ORs. For each PR component, any OR component which exactly matched or had the highest pairwise Tanimoto similarity was regarded as the true reactant corresponding to the PR. After all PRs were compared, the remaining ORs were then designated as reagents. In general, the structural similarity between reactants and reagents is quite low; reagents can easily be distinguished and removed from the reactant list.

### 2.3. Reaction Knowledge Graph

Gryzbowski et al. [38] constructed directed complex networks using known organic chemical reactions, in which the nodes refer to the chemical substances (either reactants or products) and the directed edges correspond to the chemical reactions in which the substances are involved. In current study, a similar network was constructed based on the combined Pistachio and USPTO datasets. The chemical reaction network was built with NetworkX (Version 2.6.2) [48]. The network file (GraphML format) generated from the USPTO dataset after clearing can be found in https://github.com/jidushanbojue/YaSAScore/data, (accessed on 20 December 2021). From this network, the SRP modelling dataset was generated. 

### 2.4. Synthesis Accessibility Prediction Model

**Figure 5:**
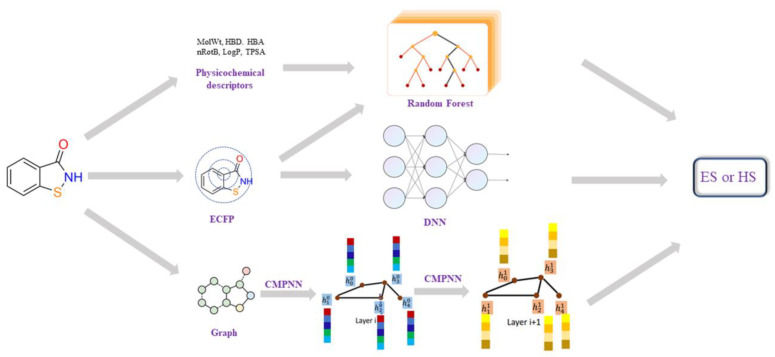
The workflow of different synthesis accessibility prediction models, including: (1) Random Forest model using the physicochemical descriptors; (2) DNN models using ECFP4; (3) graph convolution neural-network-based model using molecular 2D graph structure. Each node (atom) of a graph (molecule) is represented by a feature vector of 133 dimensions, including ‘atomic number’, ‘degree’, ‘formal charge’, ‘number of hydrogens’, ‘hybridization’ and ‘chiral tag’. Then, these node features, together with edge (bond) information, are processed by a Communicative Message Passing Neural Network (CMPNN) to generate a molecular feature.

Several model building methods were employed to train on the SRP dataset. The workflow of different models was shown in Figure 5. A classic machine learning algorithm Random Forest [49,50] was built, using some physicochemical descriptors as input. The fully connected deep neural network (DNN) [51,52] models use the 2048-length bit string Extended-Connectivity Fingerprints (ECFP4, here with a maximum searching depth of 2) as input. The DNN classifier was trained using Keras with Tensorflow as the back end, the RMSprop optimizer was used, and binary cross-entropy was chosen as the loss function. The learning rate was decayed on plateau by a factor of 0.5. The optimal combination of parameters for the model was searched based on the model’s performance on the validation set. 

A graph convolution neural-network-based model was also built, using a molecular 2D graph structure as input. Here, the modified message-passing neural network, CMPNN [41], was employed to construct the graph neural network (as shown in Figure 5). The CMPNN classifier was trained using default parameters, as in the original literature [41]. Additionally, the performance of several existing SA models, such as SYBA, SCScore and SAScore, were also examined on our dataset for the purposes of comparison. The hyper-parameters of those models and more training details can be found in the Supporting Materials (Figures S1–S5).

### 2.5. Performance Evaluation

The performance of the SA prediction models was evaluated by three different metrics: the classification accuracy (ACC), Matthews correlation coefficient (MCC) and area under the ROC curve (ROC-AUC)

(1) $$\mathrm{ACC}=\frac{\mathrm{TP}+\mathrm{TN}}{\mathrm{TP}+\mathrm{TN}+\mathrm{FP}+\mathrm{FN}}$$

(2) $$\mathrm{MCC}=\frac{\mathrm{TP}*\mathrm{TN}-\mathrm{FN}*\mathrm{FP}}{\sqrt{\left(\mathrm{TP}+\mathrm{FN}\right)\left(\mathrm{TP}+\mathrm{FP}\right)\left(\mathrm{TN}+\mathrm{FN}\right)\left(\mathrm{TN}+\mathrm{FP}\right)}}$$

where true positive (TP) refers to the true ES, and true negative (TN) refers to the true HS. ACC represents the percentage of correctly classified samples, regardless of their predicted classes. MCC is mainly used to measure binary classification problems, and takes TP, TN, FP and FN into comprehensive consideration. This is a relatively balanced indicator and can also be used in the case of unbalanced samples. The value of MCC ranges from −1 to 1, where 1 indicates that the prediction is completely consistent with the actual result; 0 indicates that the prediction result is consistent with the random prediction result; −1 indicates that the prediction result is completely inconsistent with the actual result. ROC-AUC and MCC [53] are two important metrics which form a trade-off between TP rate and FP rate over all the possible thresholds. MCC, ACC and ROC-AUC are commonly used metrics for measuring the performance of a binary classification model.

## 3. Results and Discussion

### 3.1. Data Statistics of Reaction Knowledge Graph

**Figure 6:**
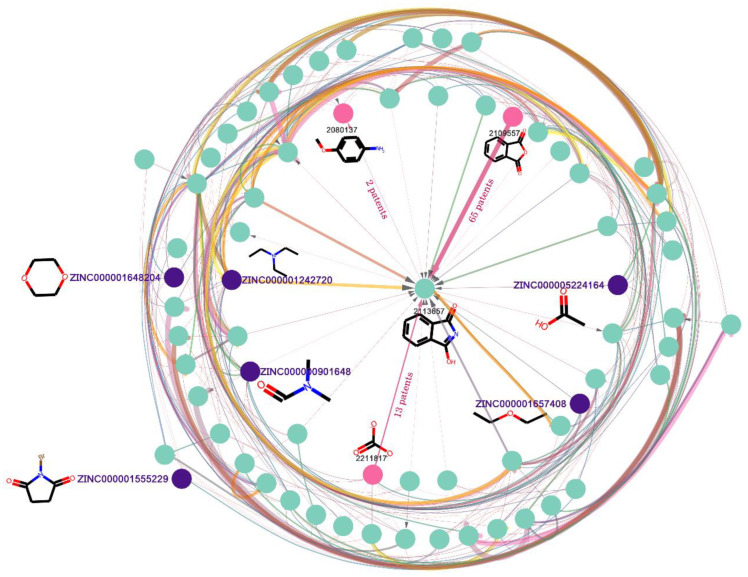
Subgraph of the reaction knowledge graph network. Nodes that begin with “ZINC” are terminal nodes as well as starting materials. Others are normal nodes that could be recognized as either the product of the starting materials, or the reactant for other products. The width of the line represents the number of chemical patents involved in the reaction. The thicker the line, the more patents use the reaction.

After going through the dataset cleaning process, substances that do not directly contribute to the reactions, such as solvents and catalysts, were removed and, finally, a refined chemical reaction network containing 2,192,740 nodes was constructed (subgraph as shown in Figure 6) and its statistical details are listed in Table 1.

As shown in Figure 6, the starting nodes of the directed edge are reactants of the reaction, and the destination nodes are the products. There are two types of node in the reaction knowledge graph: one type is the node which does not connect to any in-flux edge and only connects to out-flux edges. This is called the terminal node, which only serves as a reactant, and there are 488,220 terminal nodes (in Table 1) in the graph. Nevertheless, the terminal nodes may be limited by the dataset used to build the knowledge graph and are not necessary as starting materials. Here, we used a set of commercially available building blocks [36] from ZINC database [33] to identify the starting materials among the terminal nodes; in total, 38,664 terminal nodes (in Table 1) were identified (such as the nodes that begin with “ZINC” in Figure 6). The other type is the normal node (such as node 2113657 in Figure 6), which connects to both in-flux and out-flux edges. There is a total of 691,830 nodes in Table 1. The normal node can be recognized as either the product of the starting materials, or the reactant for other products. Last but not least, to remove some nodes with poor physical and chemical properties, we limited the molecular weight to 200~500, and LogP to 0~5. Finally, **876,074** nodes were retained, and it was named **SET1**.

**Figure 7:**
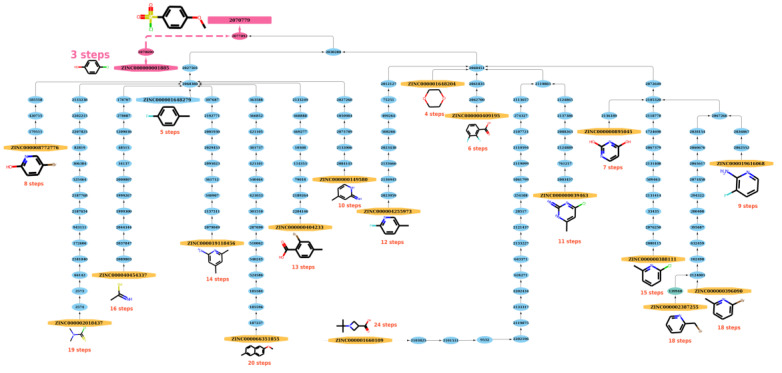
The reaction paths of product (node: 2070779) in the graph. After searching possible reaction paths, 19 reaction paths can be used to synthesize product from the respective ZINC starting materials. Among these options, at least 3 steps are needed (from ZINC000000001885 to node 2070779), so the SRP equals 3.

The path length generated by functions built in NetworkX (https://networkx.org/, accessed on 20 December 2021) between a starting material and a product on the graph can be referred as the possible reaction path to synthesize a compound. From a practical perspective, the route with the shortest steps to the product can be regarded as the best reaction route for synthesizing the product and the shortest reaction path (SRP) that was derived was used to model the study. As shown in Figure 7, after searching for possible reaction paths, there are 19 reaction paths that can be used to synthesize the product (node 2070779) from the respective ZINC starting materials. Among these optional paths, at least three steps are needed (from ZINC000000001885 to node 2070779 in Figure 7), so the SRP equals 3. See Figures S6–S8 for other, similar examples.

### 3.2. Partition Criterion of ES and HS

**Figure 8:**
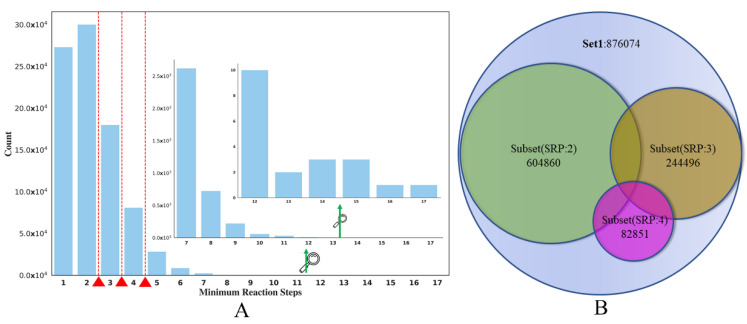
(**A**) The distribution of the **SET1**. (**B**) Splitting of **SET1** dataset into three independent subsets (SRP:2, SRP:3, SPR:4).

The main goal of the current study is to use the SRP data obtained from the reaction knowledge graph as the SA surrogate to build classification models of compound SA. The distribution of the SRP in **SET1** can be seen in Figure 8A. It seems that the distribution of SRP is quite uneven; the RS of most of the compounds is lower than 3. Here, we set the partition criterion as SRP 2, 3, 4, respectively. This means that compounds whose SRP is less than or equal to the RS cut-off were regarded as the ES class, and compounds whose SRP was greater than the cut-off were classified as the HS class. 

Here, we take the RS cut-off of 3 as an example (in Table 2). After partitioning **SET1**, 122,248 HS and 753,826 ES were produced. To create a balanced dataset for model training, a structural clustering analysis was conducted on the ES compounds to select a diverse ES compound set, which had roughly the same number of compounds as the HS class. The oetoolkit [54]-based program Flush [55] was used for clustering and the tanimoto similarity threshold, which was calculated based on Foyfi fingerprints [56], was 0.615. After clustering, a dataset containing 123,837 HS compounds was curated, in which the ratio of ES and HS compounds is roughly at 1:1, eventually generating Subset(SRP:3), 244,496 (in Figure 8B). This balanced dataset was then split into training, validation and test sets (24,450 in Table 2) with the ratio of 8:1:1. In addition, we also made full use of the dataset that was removed after clustering, and an Unbalanced-Test was also built by adding the remaining ES compounds into the balanced test set to evaluate the model performance on all compounds not included in the training set. The data statistics of other subsets are shown in Table 2 and Figure 8B.

### 3.3. Analysis of Chemical Space of the Data Set

**Figure 9:**
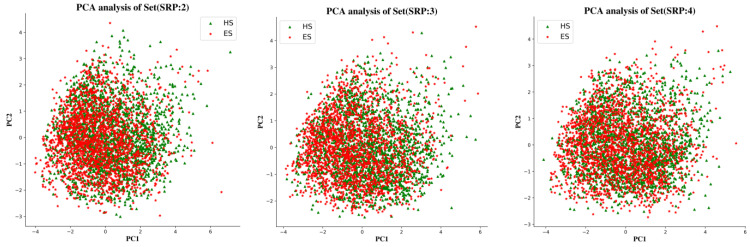
PCA analysis of the physicochemical descriptors of three subsets.

To examine the chemical space of the ES and HS compounds of each partitioning criterion, a principal component analysis (PCA) was carried out on the whole ES and HS compound sets based on six physicochemical descriptors, calculated using the RDkit package, i.e., molecular weight (MW), topological polar surface area (TPSA), number of rotatable bonds (RTB), number of H bond donors (HBD), and number of H bond acceptors (HBA). The dimensionality of the input space was reduced by PCA to the top two components, which explained 80% of the variance in the data. Figure 9 shows that the chemical spaces of three subsets generated by different portioning criteria are basically identical. There was also no significant difference in the distribution of ES and HS datapoints in each Subset.

**Figure 10:**
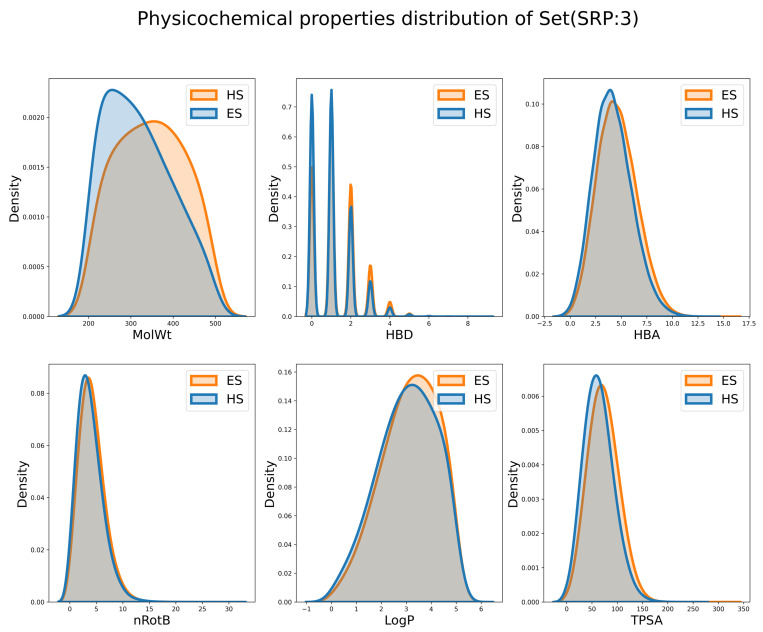
The distribution of physicochemical descriptors on Subset(SRP:3).

In addition, to explore whether the ES and HS can be distinguished by simple physicochemical properties, the distribution of individual properties is shown in Figure 10, Figures S9 and S10. It is observed that the difference in distribution on MW is the largest and the differences for other properties are minor. This observation is consistent with the common-sense assumption that the HS compounds usually have a larger RS and tend to have a larger MW. However, are those descriptors good enough to distinguish the ES and HS compounds? To answer this question, Random Forest [49,50] (RF) and deep forward network [51,52] (DNN) classifiers employing these descriptors were built, and their results are shown in Table 3. The accuracy of the RF classifier (RF-PCD in Table 3) was 0.592, 0.588 and 0.583 in different subsets. The accuracy of the DNN classifier (DNN-PCD) was 0.511, 0.584 and 0.565 in different subsets. These barely satisfactory results demonstrate that the performance of these physicochemical properties, either alone or collectively, when separating ES and HS molecules, is still suboptimal.

### 3.4. Evaluation of Model Performance

Various predictive models were built on the three subsets. For CMPNN and DNN model, the optimal parameters were determined based the performance of the validation set. Table 3 shows that, among all the models in each balanced test set, the CMPNN model always achieved the best results. It is worth mentioning that the SYBA-2 model was retrained on our own training set using the SYBA algorithm, Among the indicators (ROC AUC, ACC and MCC in Table 3), compared to other existing synthesis accessibility scoring schemes, our results show that CMPNN has a better performance than SYBA-2, albeit marginally, and outperforms SAScore and SCScore in each balanced test set.

**Figure 11:**
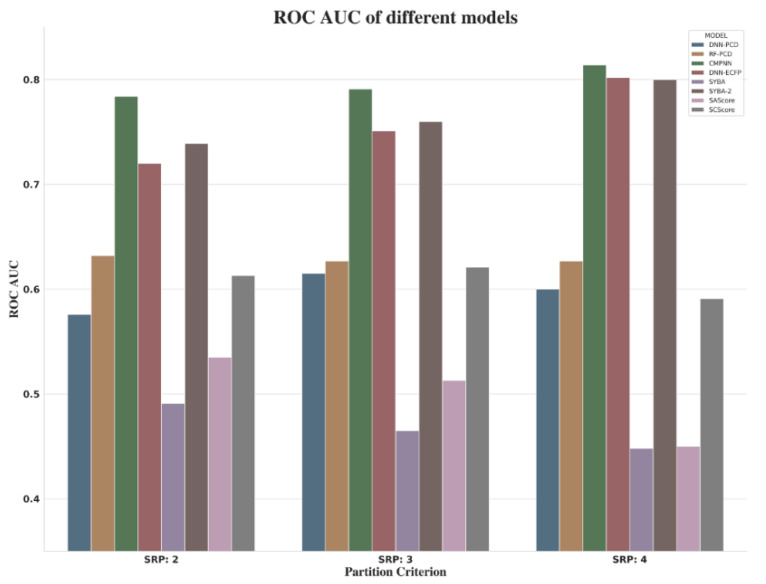
ROC AUC of different models.

When focusing on the comprehensive indicators (ROC AUC) of different models shown in Figure 11, it was observed that, as the value of RSP increases, so does the performance of CMPNN, DNN-ECFP, and SYBA. However, when choosing SRP:4, a small amount of data was required for modeling (about 80k). Due to the imbalanced nature of **SET1**, as well as the performance of models and consultancy with synthetic chemists, by asking them to inspect some examples with associated synthesis steps, we suggested SRP:3 as the threshold to distinguish ES and HS compounds. 

To gain a full picture of the model performance, different classifiers were tested on a much larger and extremely unbalanced test set. The results for every model are shown in Table 4. The ROC-AUC value of CMPNN was almost the same as that of the balanced test set and was still the best model, while the DNN-ECFP model ranked as the second-best model. The performance of the retrained SYBA-2 model decreased. It is worth mentioning that the SYBA and SAScore model have an extremely high ACC score, but their performance on ROC-AUC (0.542) and MCC (−0.001) is very poor and close to the random prediction results, which suggests that these models tend to classify all unbalanced subsets compounds as ES class, and have difficulty identifying HS molecules. This result highlights that the ACC is not actually a suitable criterion for measuring model performance in an unbalanced dataset.

These results suggest that the structural complexity of a molecule could not correlate with the actual reaction step data. Models built with actual reaction step data may better reflect the SA. Conceptually, SAScore and SYBA methods differ from the method used in the present work. The SCScore model was built on the reaction data, but only considered the relationship between reactant–product pairs. Unlike the SAScore and SYBA models, in which the datasets were selected based on structural complexity, our model is built on the true reaction step data and represents another type of SA model.

## 4. Conclusions

In the present work, we have developed predictive models for quantifying synthesis accessibility based on a chemical reaction network constructed on the USPTO and Pistachio reaction datasets. In contrast to existing SA methods, which were built based on compound complexity, we used the shortest reaction path of a product compound, which was obtained by carrying out a depth-first search of a chemical reaction network, as the surrogate synthesis accessibility. Compounds were designated as either ES or HS classes depending on their minimum synthesis steps, and two SA prediction models were built using deep learning/machine learning algorithms. We compared these SA-scoring functions with existing SA scoring schemes, such as SYBA, SCScore, SAScore. The graph convolution neural network model CMPNN outperforms the existing SA scores. It is expected that building SA prediction models based on historical reaction data could be an interesting future direction for quantitatively assessing molecular SA. With more reaction data and a bigger reaction network, the SA prediction model could be further improved.

## Figures and Tables

**Table 1:** Statistics in the reaction knowledge graph.

Products	1,368,588
Terminal node	488,220
Normal node	691,830
Starting material	38,664
Reactant + Product (network node)	2,192,740
Agent (not network node)	20,286
Reactant + Product + Agent	2,213,026

**Table 2:** ES and HS of different partition criterion.

SRP	HS	Balanced ES	ES-All	Similarity Cut-Off for Clustering	Balanced-Test	Unbalanced-Test
2	302,430	310,790	573,644	0.35	60,486	323,340
3	122,248	123,837	753,826	0.615	24,450	654,439
4	41,066	41,785	835,008	0.655	8286	801,509

**Table 3:** The performance of different models on the balanced test set.

Partition Criterion	MODEL	AUC	ACC	MCC
SRP:2	DNN-PCD^1^	0.576	0.511	0.044
SRP:2	RF-PCD^2^	0.632	0.592	0.183
SRP:2	CMPNN	**0.784**	**0.711**	**0.423**
SRP:2	DNN-ECFP^3^	0.72	0.667	0.333
SRP:2	SYBA	0.491	0.505	0.02
SRP:2	SYBA-2^4^	0.739	0.668	0.343
SRP:2	SAScore	0.535	0.501	−0.003
SRP: 2	SCScore	0.613	0.55	0.128
SRP:3	DNN-PCD^1^	0.615	0.584	0.168
SRP:3	RF-PCD^2^	0.627	0.588	0.177
SRP:3	CMPNN	**0.791**	**0.715**	**0.434**
SRP:3	DNN-ECFP^3^	0.751	0.687	0.373
SRP:3	SYBA	0.465	0.496	−0.012
SRP:3	SYBA-2^4^	0.76	0.69	0.382
SRP:3	SAScore	0.513	0.5	−0.011
SRP: 3	SCScore	0.621	0.543	0.116
SRP:4	DNN-PCD^1^	0.6	0.565	0.132
SRP:4	RF-PCD^2^	0.627	0.583	0.168
SRP:4	CMPNN	**0.814**	**0.733**	**0.466**
SRP:4	DNN-ECFP^3^	0.802	0.732	0.465
SRP:4	SYBA	0.448	0.491	−0.061
SRP:4	SYBA-2^4^	0.8	0.727	0.453
SRP:4	SAScore	0.45	0.512	−0.021
SRP:4	SCScore	0.591	0.517	0.082

DNN-PCD^1^: DNN classifier built according to physicochemical descriptors. RF-PCD^2^: RF classifier built on physicochemical descriptor. DNN-ECFP^3^: DNN classifier built on ECFP4 descriptor. SYBA-2^4^: retrained the SYBA model on our own dataset.

**Table 4:** The performance of different models on an unbalanced test set.

Partition Criterion	MODEL	AUC	ACC	MCC
SRP:2	CMPNN	**0.764**	0.666	**0.236**
SRP:2	DNN-ECFP	0.734	0.679	0.215
SRP:2	SYBA	0.562	0.88	0.041
SRP:2	SYBA-2	0.677	0.668	0.151
SRP:2	SAScore	0.542	**0.906**	−0.001
SRP:2	SCScore	0.538	0.273	0.049
SRP:3	CMPNN	**0.74**	0.592	**0.096**
SRP:3	DNN-ECFP	0.736	0.655	**0.096**
SRP:3	SYBA	0.569	0.944	0.027
SRP:3	SYBA-2	0.694	0.651	0.079
SRP:3	SAScore	0.569	**0.981**	0.002
SRP:3	SCScore	0.584	0.229	0.033
SRP:4	CMPNN	0.733	0.65	0.051
SRP:4	DNN-ECFP	**0.762**	0.633	**0.058**
SRP:4	SYBA	0.581	0.951	0.016
SRP:4	SYBA-2	0.737	0.616	0.05
SRP:4	SAScore	0.587	**0.995**	−0.001
SRP:4	SCScore	0.616	0.213	0.021

## Data Availability

The Pistachio dataset was a commercially available chemical reaction database. Filbert, NameRxn and HazELNut were used for atom-mapping and reaction classifications under license from NextMove. The detailed hyper-parameters of all models can be found in the Supplementary Materials. The scripts for template extraction, generation of a chemical reaction network, and model building can be found in the GitHub repository https://github.com/jidushanbojue/YaSAScore (accessed on 20 December 2021).

## Supplementary Materials

The following supporting information can be downloaded, Table S1: The essential hyper-parameters of models; Figure S1: Accuracy log of DNN-Physicochemical model; Figure S2: Loss log of DNN-Physicochemical model; Figure S3: Accuracy log of DNN-ECFP model; Figure S4: Loss log of DNN-ECFP model; Figure S5: ROC-AUC log of CMPNN model; Figure S6: The reaction paths of product (node: 56596) in the graph; Figure S7: The reaction paths of product (node: 958968) in the graph; Figure S8: The reaction paths of product (node: 535835) in the graph; Figure S9: Physicochemical properties distribution of Subset (SRP:2); Figure S10: Physicochemical properties distribution of Subset (SRP:4).

## Sample Availability

All the mentioned reactions can be retrieved from USPTO and Pistachio database. All the mentioned compounds can be bought from chemical suppliers.

## Abbreviations

PRs: Predicted Reactants; ORs, Original Reactants; ES, Easy to Synthesize; HS, Hard to Synthesize; NOC, Network Of Chemistry; SRP, Shortest Reaction Path.
