# Supplementary material for: Prediction of Compound Synthesis Accessibility Based on Reaction Knowledge Graph
Source: Molecules. 2022 Feb 3;27(3):1039. doi: 10.3390/molecules27031039 (PMC8838603; doi:10.3390/molecules27031039)
Supplement: Supplementary file 1 [file molecules-27-01039-s001.zip › molecules-1526896-supplementary.pdf]

Table S1. The essential hyper-parameters of models

	Machine learning (ML) method	Availability	Referenced from	Parameters
CMPNN	Graph model -- classification	https://github.com/jidushanbojue/YaSAScore/CMPNN-master	https://github.com/SY575/CMPNN	Framework: CMPNN<br>Epochs: 30<br>Num_folds: 1<br>Batch_size: 50<br>Ensemble_size: 1<br>Hidden_size: 300<br>Init_lr:1e-4<br>Final_lr: 1e-4<br>Max_lr: 1e-3<br>Depth(Number of message passing steps): 3<br>Dropout: 0.0<br>Activation: Relu<br>FFN_Num_layers: 2
DNN-ECFP	DNN -- classification	https://github.com/jidushanbojue/YaSAScore/DNN_model		Framework: Tensorflow<br>Dense Layers： 3: (2048, 512, 2)<br>Regularization: L2(0.0001)<br>Optimizer: RMSprop<br>Epochs: 500<br>EarlyStop patience: 80<br>BatchNormalization: True

SYBA	Naïve Bayers -- classificatioin	https://github.com/jidushanbojue/YaSAScore/sascore_scscore_syba_syba2_model	https://github.com/lich-uct/syba	Framework: SYBA<br>Statistics file of molecular fragments: https://github.com/jidushanbojue/YaSAScore/sascore_scscore_syba_syba2_model/syba/resources/syba4.csv.gz
SYBA-2	Naïve Bayers -- classificatioin	https://github.com/jidushanbojue/YaSAScore/sascore_scscore_syba_syba2_model	https://github.com/lich-uct/syba	Framework: SYBA<br>Statistics file of molecular fragments: https://github.com/jidushanbojue/YaSAScore/sascore_scscore_syba_syba2_model/syba/resources/syba_ES_cluster_HS_train_val.csv
SAScore	N/A	https://github.com/jidushanbojue/YaSAScore/sascore_scscore_syba_syba2_model	https://github.com/rdkit/rdkit/blob/master/Contrib/SA_Score/sascorer.py	Framework: SAScore (RDKit)
SCScore	DNN -- regression	https://github.com/jidushanbojue/YaSAScore/sascore_scscore_syba_syba2_model	https://github.com/connorcoley/scscore	Framework: SCScore

Note: 1. The same data set were used by all models above. For DNN-ECFP model and CMPNN model, after retrained and update the corresponding models, the optimal model parameters were shown in Table S1.

2. SAScore model was implemented by adopted the script built in RdKit (https://github.com/rdkit/rdkit/blob/master/Contrib/SA_Score/sascorer.py)

3. SCScore model was implemented by adopted the script built in SCScore (https://github.com/connorcoley/scscore)

4. SYBA model was implemented by adopted the script built in SYBA (https://github.com/lich-uct/syba)

5. SYBA-2 model was retrained by generating the new statistics data of molecular fragments (syba_ES_cluster_HS_train_val.csv) on new dataset, and then file was used to replace the original syba4.csv.gz in https://github.com/jidushanbojue/YaSAScore/sascore_scscore_syba_syba2_model/syba/resources/.

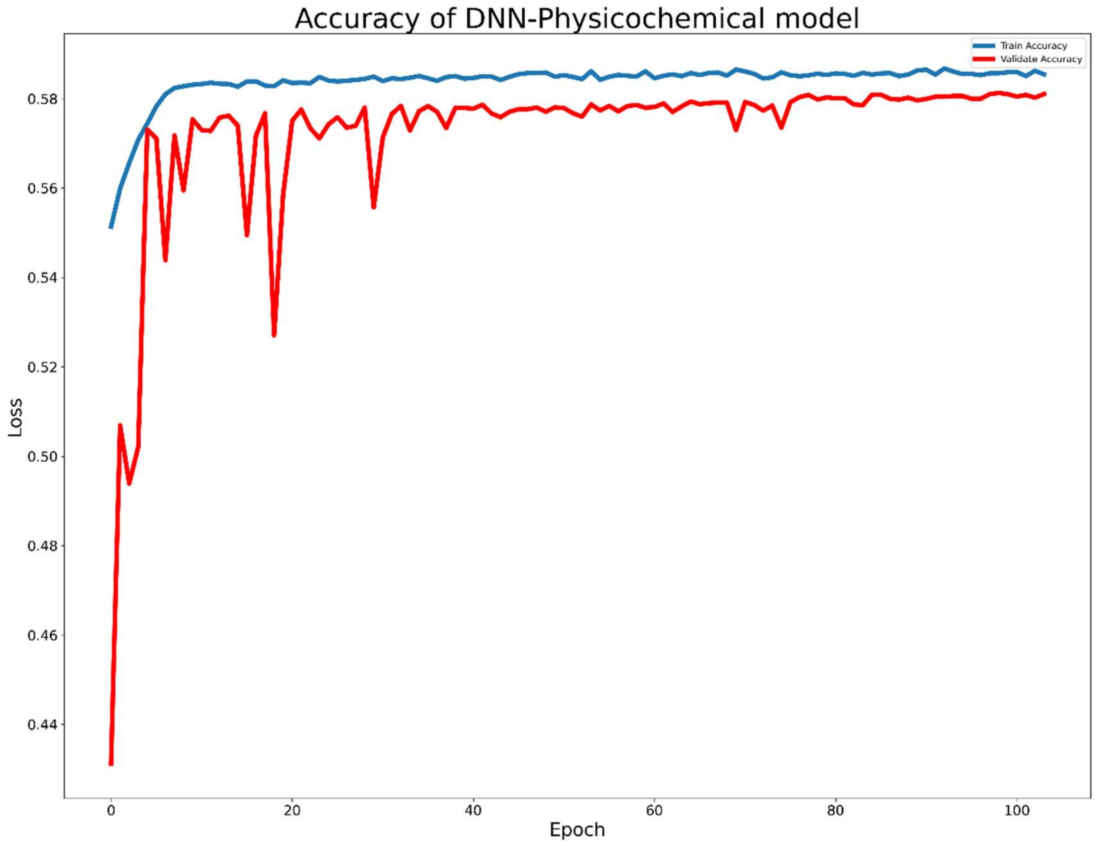

Figure S1. Accuracy log of DNN-Physicochemical model

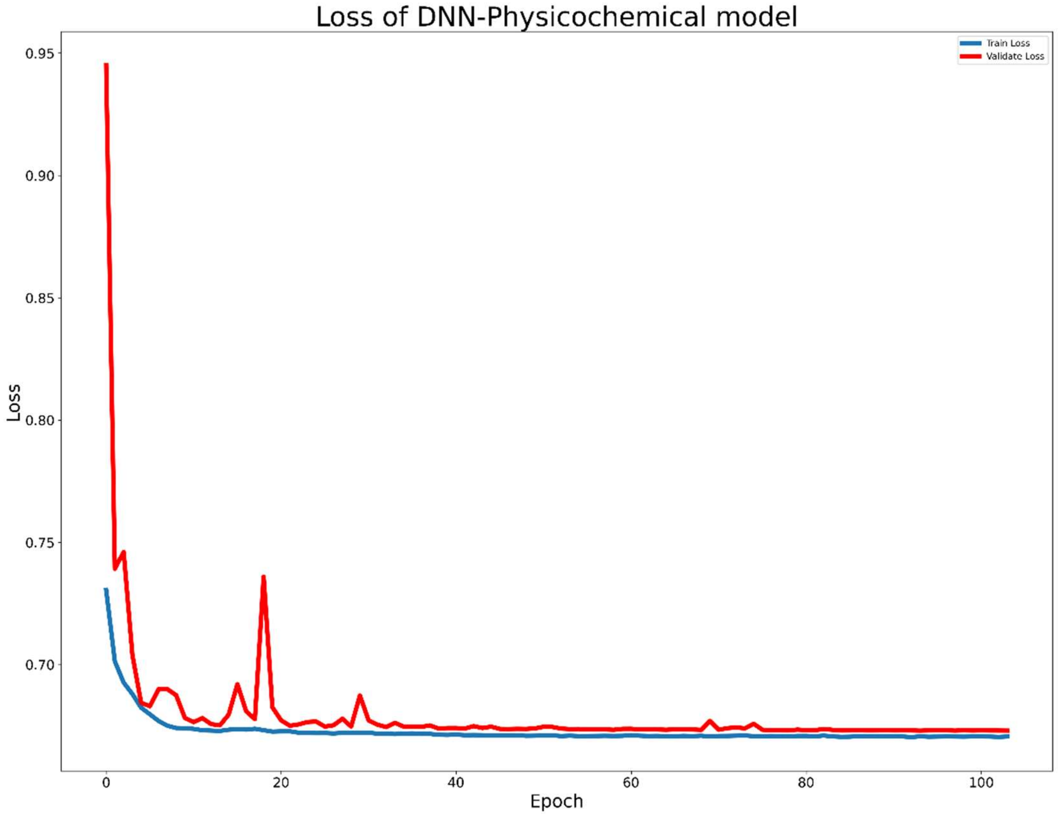

Figure S2. Loss log of DNN-Physicochemical model

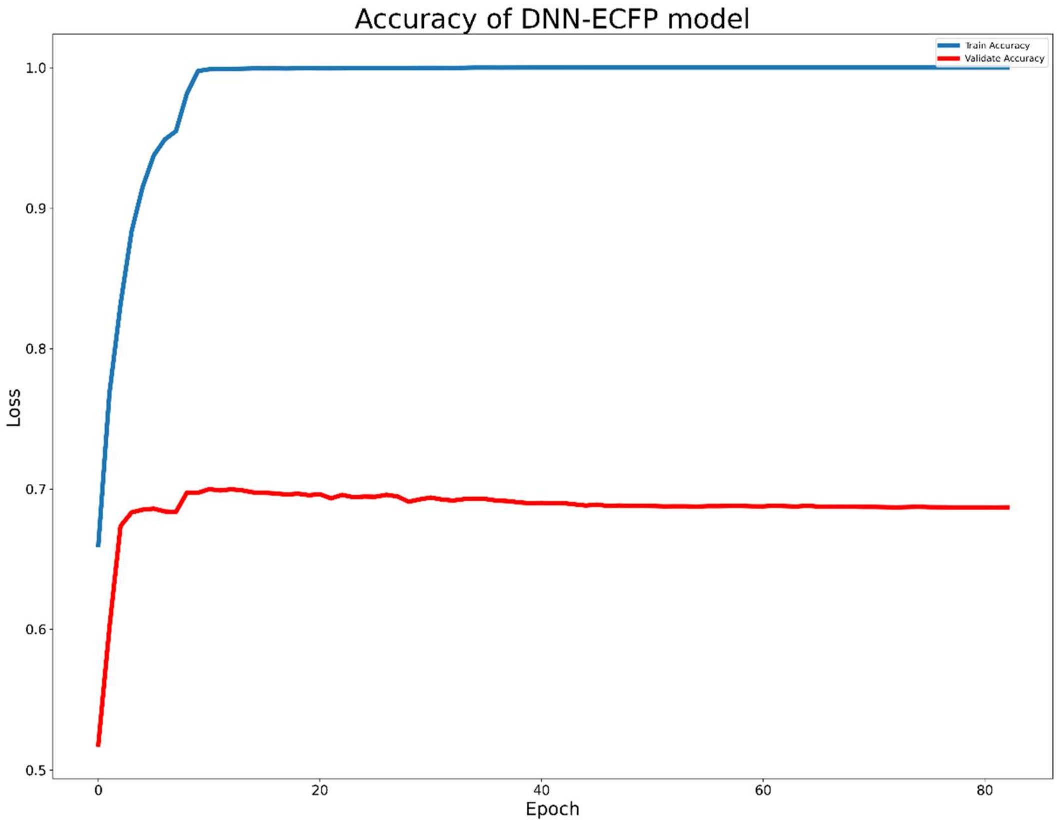

Figure S3. Accuracy log of DNN-ECFP model

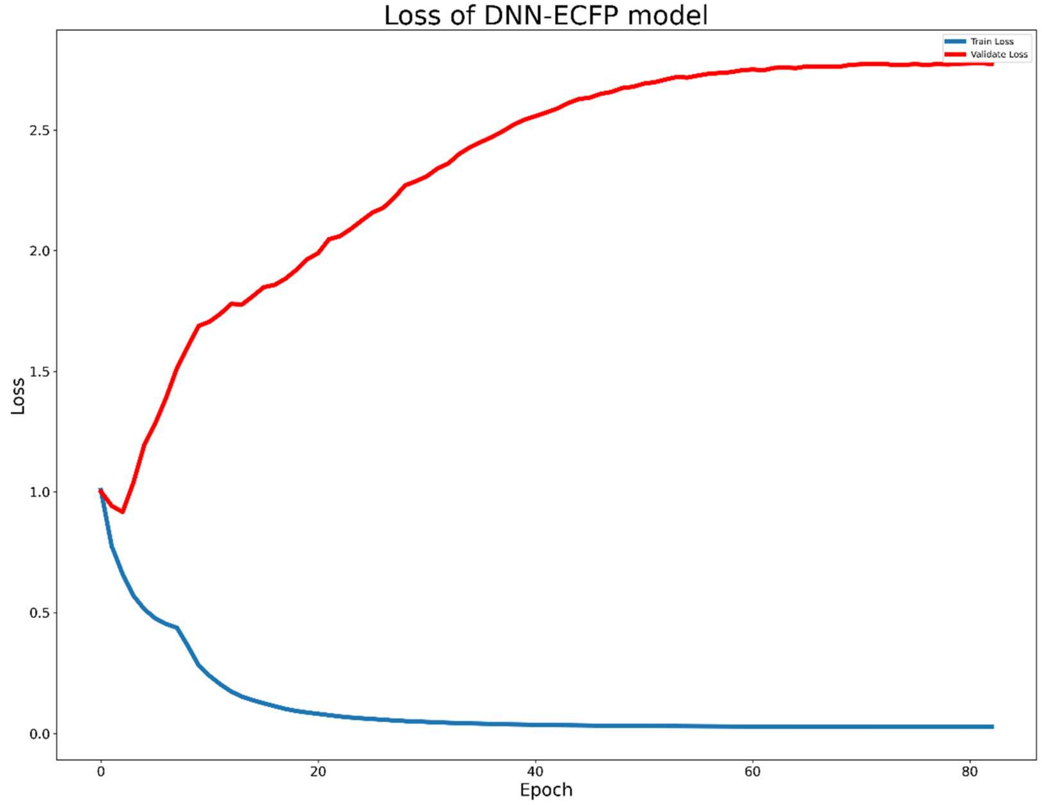

Figure S4. Loss log of DNN-ECFP model

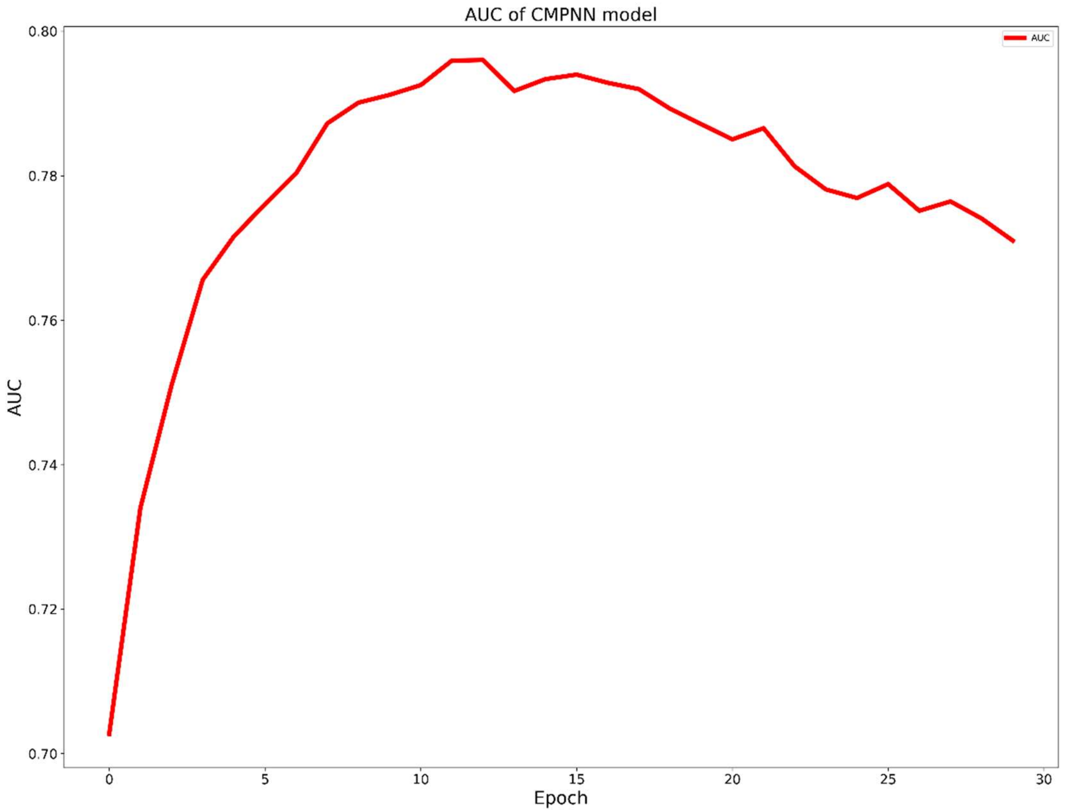

Figure S5. ROC-AUC log of CMPNN model

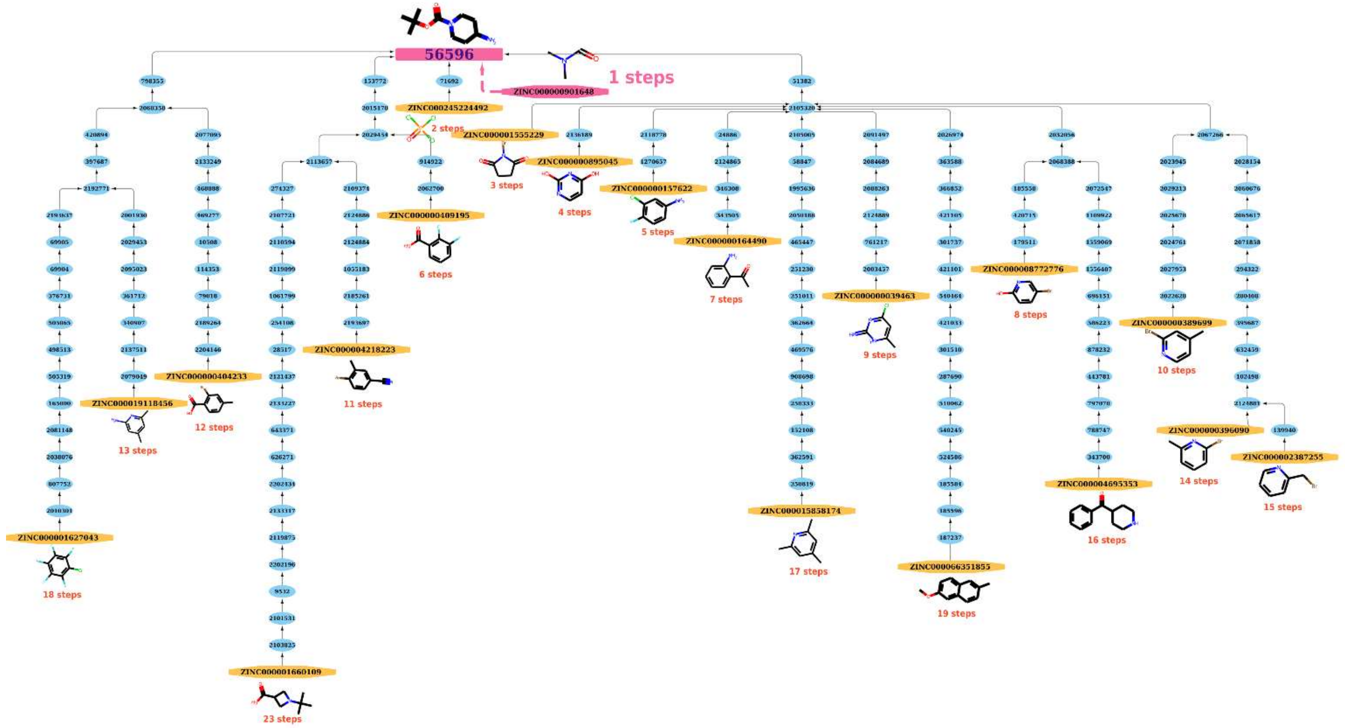

Figure S6. The reaction paths of product (node: 56596) in the graph. After searching possible reaction path, there are 20 reaction paths that can be used to synthesize product from respective ZINC starting materials. Among these options, it takes at least **1** steps (from ZINC000000901648 to node 56596)

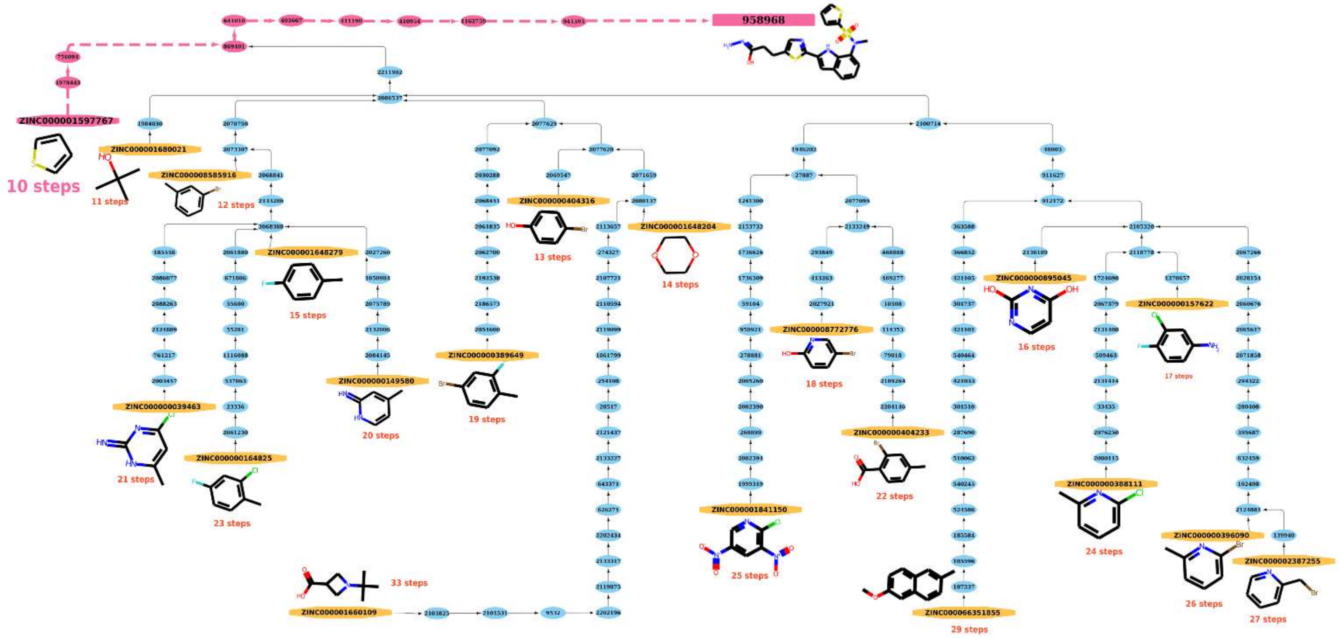

Figure S7. The reaction paths of product (node: 958968) in the graph. After searching possible reaction path, there are 19 reaction paths that can be used to synthesize product from respective ZINC starting materials. Among these options, it takes at least **10** steps (from ZINC000001657408 to node 958968)

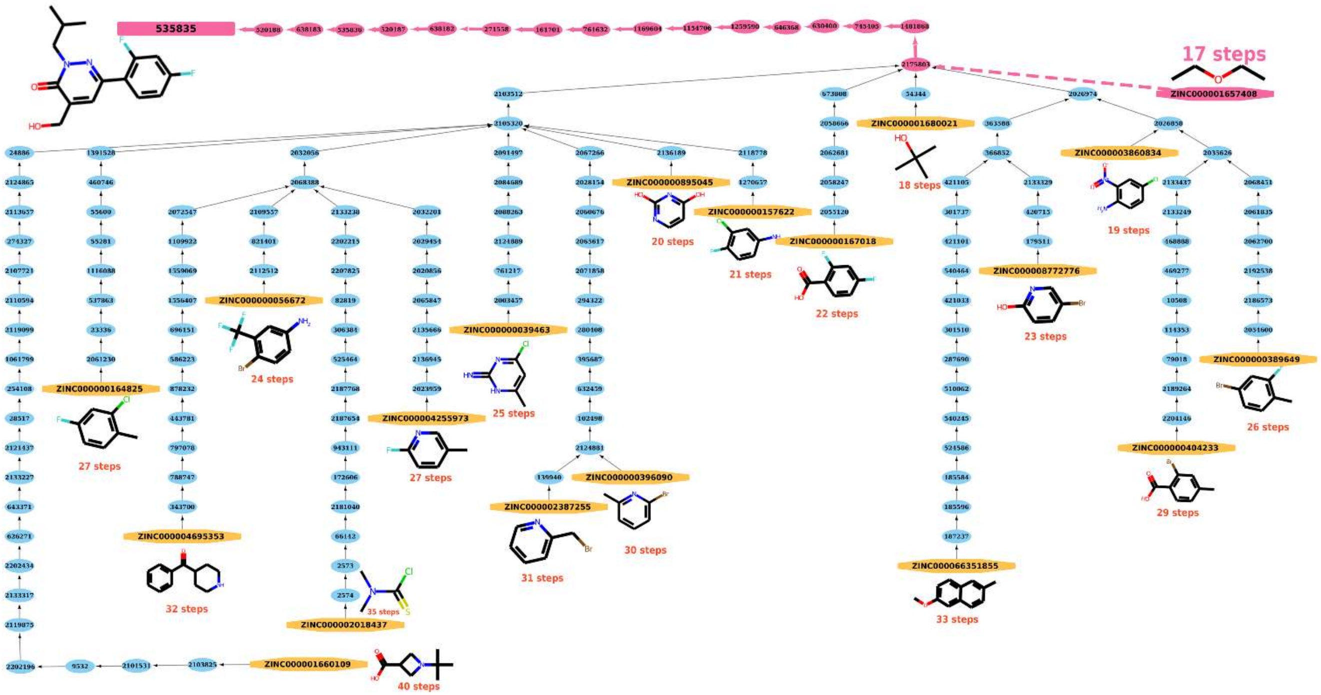

Figure S8. The reaction paths of product (node: 535835) in the graph. After searching possible reaction path, there are 19 reaction paths that can be used to synthesize product from respective ZINC starting materials. Among these options, it takes at least **17** steps (from ZINC000001657408 to node 535835)

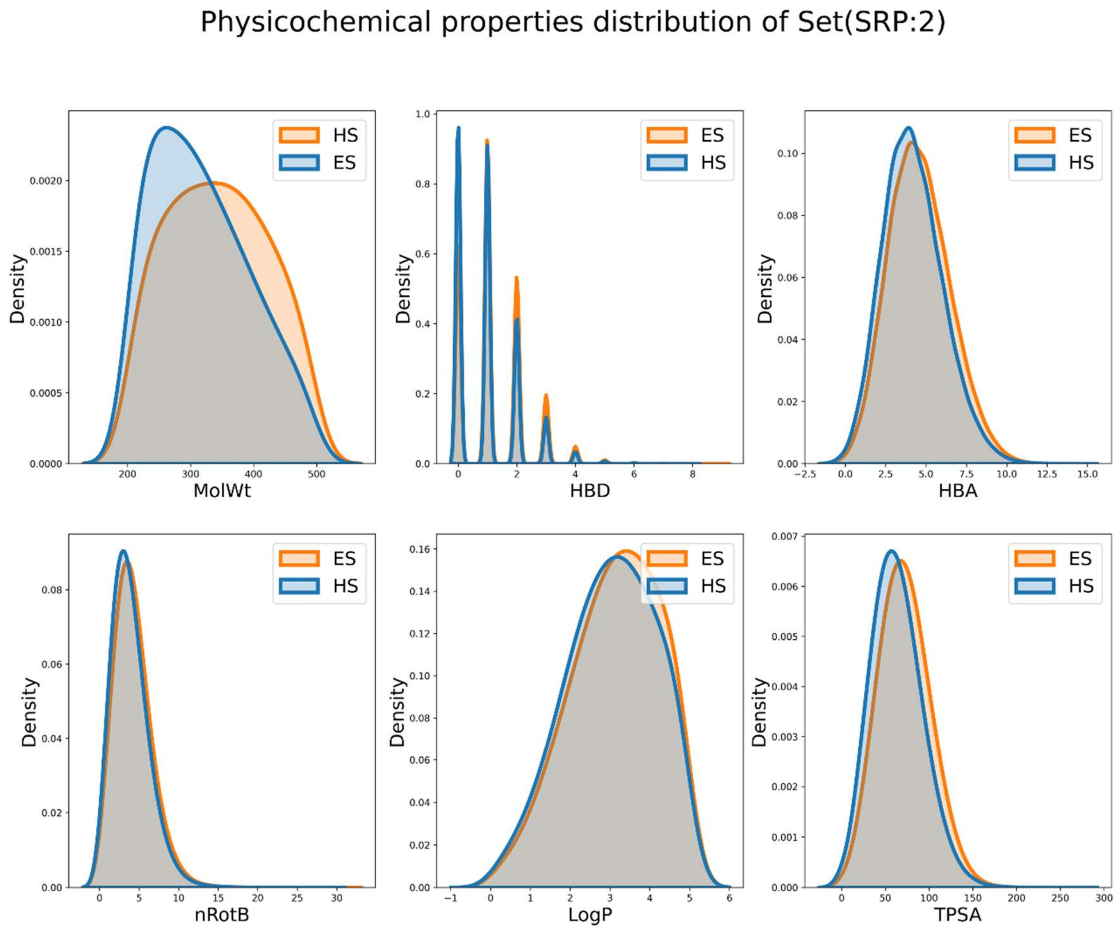

Figure S9. Physicochemical properties distribution of Subset (SRP: 2)

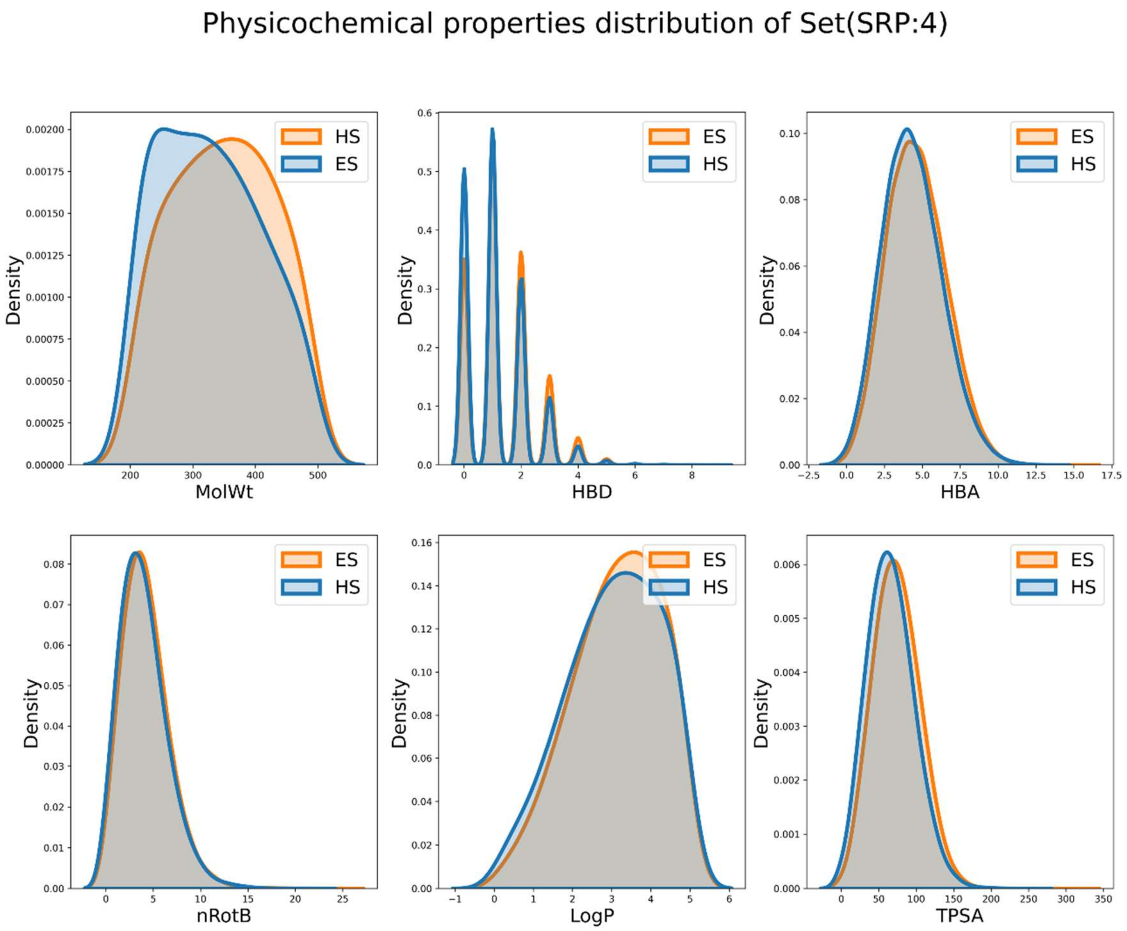

Figure S10. Physicochemical properties distribution of Subset (SRP: 4)
